# Maternal weight during pregnancy and risk of childhood acute lymphoblastic leukemia in offspring

**DOI:** 10.1038/s41375-025-02517-6

**Published:** 2025-01-26

**Authors:** Jiaye Liu, Elham Kharazmi, Qunfeng Liang, Yafei Chen, Jan Sundquist, Kristina Sundquist, Mahdi Fallah

**Affiliations:** 1 Risk Adapted Prevention Group, Division of Primary Cancer Prevention, German Cancer Research Center (DKFZ), Heidelberg, Germany; 2 Medical Faculty Heidelberg, Heidelberg University, Heidelberg, Germany; 3 Center for Primary Health Care Research, Department of Clinical Sciences, Lund University, Malmö, Sweden; 4 University Clinic Primary Care Skåne, Region Skåne, Sweden; 5 Institute of Primary Health Care (BIHAM), University of Bern, Bern, Switzerland

**Keywords:** Risk factors, Cancer epidemiology, Acute lymphocytic leukaemia

## Abstract

In addition to biological factors, maternal exposures during pregnancy can contribute to leukemogenesis in offspring. We conducted a population-based cohort study in Sweden to investigate the association between risk of acute lymphoblastic leukemia (ALL) in offspring and maternal anthropometrics during pregnancy. A total of 2,961,435 live-born singletons during 1983–2018 were followed from birth to ALL diagnosis, end of age 18, or end of 2018. 1388 children were diagnosed with ALL (55.6% boys). We observed an increased risk of ALL among daughters of overweight/obese mothers in early pregnancy [Body mass index (BMI) ≥ 25 kg/m^2^; Standardized incidence ratio (SIR) = 1.4, 95% CI: 1.2–1.6] compared with the risk in daughters of mothers with normal BMI. This association was not found in their sons (SIR = 1.0, 95% CI: 0.9–1.1). Similar results were found for the association between ALL and maternal BMI before delivery. We did not find an association between low or high gestational weight gain (GWG) and risk of ALL (both SIRs = 1.0) in male/female offspring. These suggest that maternal overweight/obesity are important risk factors for childhood ALL in daughters, whereas GWG is not associated with risk of ALL. Further research on this mother-daughter association may shed light on a possible sex hormone/chromosome-related etiology of ALL.

## Introduction

Acute lymphoblastic leukemia (ALL) represents the most common childhood malignancy, accounting for approximately 25% of all childhood cancers and 80% of childhood leukemias [1]. The overall incidence of childhood cancer, including ALL, has consistently increased since 1975 (ref. [2]). Despite a growing amount of research, the etiology of ALL remains not fully understood and effective primary prevention measures are lacking.

The prevailing theory suggests that ALL may originate in utero, as evidenced by the detection of acquired somatic mutations at birth in children with leukemia [3, 4]. This indicates that maternal exposures associated with genetic alterations during pregnancy may play a role in the development of ALL. The rising prevalence of obesity among women of childbearing age has garnered significant attention [5]. Studies have shown an increased risk of several cancers including breast and endometrial cancer in obese women [6, 7] through potential mechanisms, such as promoted mitosis caused by high endogenous estrogen levels, elevated insulin-like growth factor-1 (IGF-1) levels caused by hyperinsulinemia, and chronic inflammation stimulation [8, 9]. However, the association between maternal obesity and childhood cancer risk remains understudied.

Previous studies have reported an increased risk of childhood ALL in children with high birth weight [10], which may be linked to IGF-1 levels [11]. As maternal obesity and excessive weight gain during pregnancy have been shown to promote fetal growth [12, 13], these conditions may increase the risk of ALL associated with high birth weight. Moreover, maternal anthropometrics during pregnancy are easier to monitor and intervene with than birth weight, making them a potentially modifiable risk factor. These highlight the need for further research on the potential role of maternal anthropometrics during pregnancy in the development of ALL in offspring.

Maternal pre-pregnancy obesity has been associated with a higher risk of childhood leukemia [14–16], but the role of maternal weight in early pregnancy and before delivery in the development of ALL remains unclear. The few studies focusing on the relationship between gestational weight gain (GWG) and leukemia had case-control design with limited study power and produced inconsistent results [14, 16–18].

In our study, we aimed to identify and quantify the risk of childhood ALL based on GWG and maternal weight and body mass index (BMI) in early pregnancy and before delivery in a nationwide cohort of women in Sweden with nearly 3 million pregnancies.

## Methods

The nationwide cohort study protocol was approved by the Regional Ethical Review Board in Lund, Sweden. No informed consent was required for register-based studies according to Swedish laws. This study followed the Strengthening the Reporting of Observational Studies in Epidemiology (STROBE) reporting guideline for observational studies.

### Study population

**Fig. 1:**
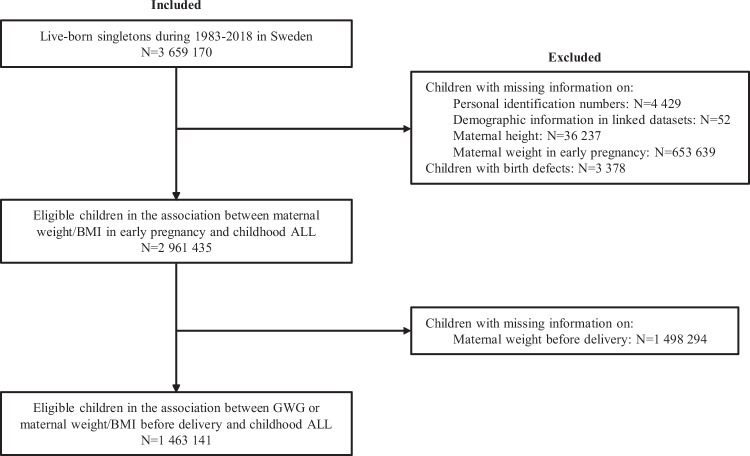
Flowchart of the study population and reasons for exclusion. ALL Acute lymphoblastic leukemia, BMI Body mass index, GWG Gestational weight gain.

All live-born children from 1983 to 2018 in Sweden (*N* = 3,659,170) were included (Fig. 1). We linked individuals from the Swedish Medical Birth Register with six other Swedish national registers (including the Cancer Registry, the National Patient Register, the National Population Register, the Multi-generation Register, the Cause of Death Register, and national censuses) using unique personal identification numbers replaced by pseudonymized serial numbers (Details in eMethods in Supplement). We excluded children without information on a personal identification number (*N* = 4429), demographic information in linked datasets (*N* = 52), maternal weight in early pregnancy (*N* = 653,639), and maternal height (*N* = 36,237). We also excluded 3378 children with birth defects, as children with birth defects, especially Down syndrome, have been reported to have a higher risk of ALL [19]. A total of 2,961,435 children were included in the study of the association between maternal weight in early pregnancy and ALL. We also excluded 1,498,294 children with missing information on maternal weight before delivery, and finally 1,463,141 children were included in our study on the association between children ALL and maternal weight before delivery or GWG. We followed these children from birth until the date of diagnosis of ALL, death, emigration, end of age 18, or end of 2018, whichever came first.

### Exposure

Data on maternal weight and height have been recorded in the Medical Birth Register since 1983. Height was self-reported at the first antenatal visit, in most cases during the first 12 weeks of pregnancy. Weight was measured at the mother’s first antenatal visit and on admission to the maternity ward. Information on maternal height is available for about 80% of the mothers. Weight in early pregnancy is available for 70% of the records, and weight before delivery is available for almost 80% of women before 1990 and 35% after 1993 (ref. [20]).

Maternal BMI was calculated as maternal weight in kilograms divided by height in squared meter (kg/m^2^). The classification of maternal BMI in early pregnancy was based on the standard category developed by the World Health Organization: Underweight (BMI < 18.5 kg/m^2^), normal weight (BMI 18.5–24.9 kg/m^2^), overweight (BMI 25–29.9 kg/m^2^), and obese (BMI ≥ 30 kg/m^2^). The categorization of maternal BMI before delivery was based on the aforementioned standard category values, with an addition of 4 BMI units, which is approximately equal to the average increase in BMI observed during pregnancy in our study population.

GWG was defined as the difference between pre-pregnancy weight and antepartum weight, including the weight of fetus, amniotic fluid, and placenta. As information on maternal pre-pregnancy weight was not available and weight gain in the first trimester during pregnancy was very small [21–23], we calculated GWG as the weight on admission to the maternity ward right before delivery minus the weight at the first antenatal visit. We divided GWG into ‘low’, ‘normal’, and ‘high’ groups according to two categorical methods: 1) The absolute value in kilograms (<10, 10–14.9, & ≥15); 2) Guidelines established by the Institute of Medicine (IOM) of the National Research Council in 2009, in which the definition of each GWG group is based on pre-pregnancy BMI [21]. For example, underweight mothers are classified in the ‘normal’ GWG group if their GWG is between 12.5 kg and 18 kg, whereas obese mothers are classified as ‘normal’ if their GWG is between 5 kg and 9 kg.

### Outcome

We identified childhood ALL using the International Classification of Diseases, 8th Revision (ICD-8) and Pathological Anatomical Diagnosis codes in the Swedish Cancer Registry (Supplementary eTable 1 in Supplement). We also used International Classification of Diseases for Oncology (ICD-O) codes for validation and supplementation of the cancer diagnosis.

### Covariates

**Fig. 2:**
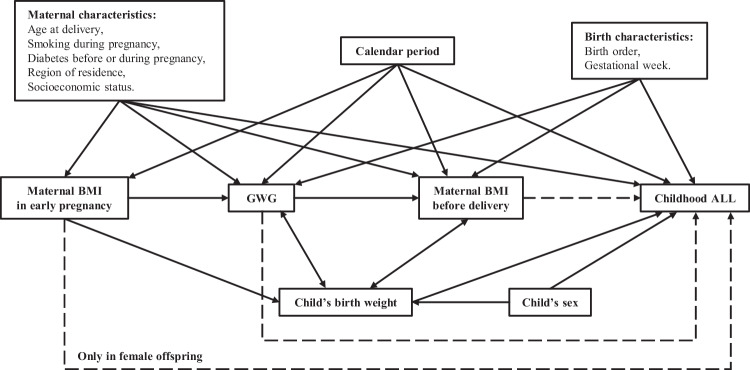
Directed acyclic graph representing associations between maternal anthropometrics during pregnancy, maternal characteristics, birth characteristics, and childhood acute lymphoblastic leukemia. ALL Acute lymphoblastic leukemia, BMI Body mass index, GWG Gestational weight gain.

Information on covariates was obtained from various Swedish registers (eMethods in Supplement). Based on extensive literature and our preliminary exploration, we identified several potentially relevant covariates in our datasets (Fig. 2). Covariates included in our adjusted analyses were child’s sex, birth year (1983–1989, 1990–1999, 2000–2009, or 2010–2018), birth weight (<2500, 2500–3499, 3500–4499, or ≥4500 g), gestational week (<37, 37–41, or ≥42 weeks), birth order (1, 2, or ≥3), and having at least one FDR with ALL (yes or no), as well as maternal socioeconomic status (farmer, blue-collar worker, high-income professional, self-employed, or unspecified), area of residence (large cities, southern Sweden, northern Sweden, or unspecified), height (<160, 160–169, or ≥170 cm), smoking during pregnancy (yes, no, or unspecified), diabetes before and during pregnancy (yes or no), and age at delivery (<25, 25–34, or ≥35 years).

To control the potential confounding effect of the baseline characteristics in our analysis, we adjusted for the demographic information, including calendar period (10-year intervals), socioeconomic status, and geographic region. Numerous studies have demonstrated a higher incidence of childhood ALL in boys compared to girls. Therefore, we controlled for child’s sex in all analyses through adjustment and stratification. Child’s birth weight may act as a potential mediator considering that birth weight may be a risk factor for childhood ALL and is strongly associated with maternal weight during pregnancy and GWG. Accordingly, we controlled for child’s birth weight through adjustment and stratification in our analysis of the association between maternal weight/BMI in early pregnancy and childhood ALL. Given the inherent inclusion of birth weight in GWG and maternal weight before delivery, we avoided over-adjustment by not further adjusting for birth weight in these analyses. In addition, birth characteristics (including birth order and gestational week) are associated with an increased risk of ALL, possibly by influencing fetal development and immune function. Therefore, we also adjusted these two factors in the association between maternal weight before delivery or GWG and risk of childhood ALL.

Women who are overweight or obese may have an elevated risk of developing diabetes, and excessive GWG is also associated with an increased risk of gestational diabetes. Both conditions have been implicated in an increased risk of childhood cancer. Therefore, we adjusted maternal diabetes before or during pregnancy in all analyses. We also adjusted our analyses for other maternal characteristics, including maternal age at delivery and smoking during pregnancy, since these have been established as risk factors for adverse birth outcomes and childhood cancers. Moreover, we additionally adjusted for maternal BMI in early pregnancy to further refine our analysis of the association between GWG and childhood ALL.

### Statistical analysis

We calculated standardized incidence ratios (SIRs) to compare the risk of ALL in offspring in different exposure groups. SIRs were calculated as the ratio of the observed to the expected number of incident cases of ALL. Expected numbers for childhood ALL were calculated by multiplying strata-specific person-years in children with underweight/overweight mothers or with inadequate/excess GWG by the corresponding strata-specific incidence rates in children with mothers of normal weight/GWG. For adjustment, we used child’s sex, decade of birth, and maternal residential area, socioeconomic status, age at delivery, history of diabetes before or during pregnancy, and smoking during pregnancy. In the analysis for maternal BMI in early pregnancy, we included information on child’s birth weight in our adjustment models. In the analysis for maternal BMI before delivery, we additionally adjusted for child’s birth order and gestational week. In the analysis for maternal weight in early pregnancy or before delivery, we also included information on maternal height based on the former adjustments. In the analysis for GWG, we additionally adjusted for maternal BMI in early pregnancy, child’s birth order, and gestational week. The Poisson distribution was assumed for the calculation of the 95% confidence intervals (CI) of the SIRs.

To examine whether the association was modified by child’s sex, as many reports have shown that boys have a higher incidence rate than girls, all analyses were stratified by sex. For further analysis, we stratified by the child’s age (0–5 or 6–18 years), child’s birth weight (<3500 or ≥3500 g), maternal smoking during pregnancy (yes or no), and maternal age at delivery (<30 or ≥30 years). For the association between GWG and childhood ALL, we additionally stratified by maternal BMI in early pregnancy (<25 or ≥25 kg/m^2^).

To assess the joint association of maternal BMI in early pregnancy and child’s sex with the risk of childhood ALL, a Cox proportional hazards model was used to incorporate the cross-product terms of these two variables to assess the multiplicative interaction. A sensitivity analysis was performed after excluding children with a family history of ALL as hereditary conditions have been estimated to account for 2%-6% of childhood ALL [24, 25].

All analyses were performed using SAS software, version 9.4 (SAS Institute Inc., Cary, NC, USA).

## Results

### Maternal weight and BMI in early pregnancy and before delivery

Of the 2,961,435 children included in the study, 1388 received a diagnosis of ALL during follow-up, of whom 772 (55.6%) were boys and 616 (44.4%) were girls. 3.6% of all children were born to underweight mothers, 64.6% to mothers with normal weight, 22.4% to overweight mothers, and 9.5% to obese mothers. The distribution of child’s gestational week, birth order, and family history of ALL, as well as maternal delivery age and diabetes, was similar in children with and without ALL, whereas the proportion of child’s high birth weight and maternal smoking during pregnancy was slightly higher in the children with ALL than in those without ALL (Table 1).

**Table 1:** Distribution of child’s and maternal characteristics in the study population.

Characteristics	Acute lymphoblastic leukemia in offspring		
	Total	No	Yes
	No. (%)	No. (%)	No. (%)
Total	2,961,435 (100.00)	2,960,047 (99.95)	1388 (0.05)
Children			
Sex			
Male	1,522,339 (51.41)	1,521,567 (51.40)	772 (55.62)
Female	1,439,096 (48.59)	1,438,480 (48.60)	616 (44.38)
Birth year			
1983–1989	521,118 (17.60)	520,804 (17.59)	314 (22.63)
1990–1999	655,829 (22.15)	655,431 (22.14)	398 (28.67)
2000–2009	855,015 (28.87)	854,565 (28.87)	450 (32.42)
2010–2018	929,473 (31.38)	929,247 (31.40)	226 (16.28)
Birth weight (g)			
<2500	86,363 (2.92)	86,325 (2.91)	38 (2.73)
2500–3499	1,249,528 (42.19)	1,249,002 (42.20)	526 (37.90)
3500–4499	1,509,114 (50.96)	1,508,361 (50.96)	753 (54.25)
≥4500	109,216 (3.69)	109,148 (3.69)	68 (4.90)
Missing	7214 (0.24)	7211 (0.24)	3 (0.22)
Gestational week			
<37	134,519 (4.54)	134,461 (4.54)	58 (4.18)
37–41	2,615,172 (88.31)	2,613,954 (88.31)	1218 (87.75)
≥42	210,761 (7.12)	210,649 (7.12)	112 (8.07)
Missing	983 (0.03)	983 (0.03)	0 (0.00)
Birth order			
1	1,144,737 (38.65)	1,144,128 (38.65)	609 (43.88)
2	963,584 (32.55)	963,134 (32.54)	450 (32.42)
≥3	542,057 (18.30)	541,807 (18.30)	250 (18.01)
Missing	311,057 (10.50)	310,978 (10.51)	79 (5.69)
Family history of ALL			
No	2,957,904 (99.88)	2,956,519 (99.88)	1385 (99.78)
Yes	3341 (0.11)	3338 (0.11)	3 (0.22)
Missing	190 (0.01)	190 (0.01)	0 (0.00)
Mothers			
BMI in early pregnancy (kg/m^2^)			
<18.5	105,899 (3.58)	105,839 (3.58)	60 (4.32)
18.5–24.9	1,911,793 (64.56)	1,910,902 (64.56)	891 (64.19)
25–29.9	662,105 (22.36)	661,794 (22.36)	311 (22.41)
≥30	281,638 (9.50)	281,512 (9.51)	126 (9.08)
BMI before delivery (kg/m^2^)			
<22.5	58,864 (1.99)	58,833 (1.99)	31 (2.23)
22.5–28.9	818,979 (27.65)	818,562 (27.65)	417 (30.04)
29–33.9	422,271 (14.26)	422,074 (14.26)	197 (14.19)
≥34	163,027 (5.51)	162,954 (5.51)	73 (5.26)
Missing	1,498,294 (50.59)	1,497,624 (50.59)	670 (48.27)
Gestational weight gain^a^			
Low	372,255 (12.57)	372,079 (12.57)	176 (12.68)
Normal	561,952 (18.98)	561,672 (18.98)	280 (20.17)
High	528,934 (17.86)	528,672 (17.86)	262 (18.88)
Missing	1,498,294 (50.59)	1,497,624 (50.59)	670 (48.27)
Delivery age (yr)			
<25	457,052 (15.43)	456,839 (15.43)	213 (15.35)
25–34	1,922,558 (64.92)	1,921,638 (64.92)	920 (66.28)
≥35	581,825 (19.65)	581,570 (19.65)	255 (18.37)
Smoking during pregnancy			
No	2,514,503 (84.91)	2,513,352 (84.91)	1151 (82.93)
Yes	388,446 (13.12)	388,245 (13.12)	201 (14.48)
Missing	58,486 (1.97)	58,450 (1.97)	36 (2.59)
Diabetes			
No	2,908,946 (98.23)	2,907,590 (98.23)	1356 (97.69)
Yes	52,489 (1.77)	52,457 (1.77)	32 (2.31)

*ALL* Acute lymphoblastic leukemia, *BMI* Body mass index, *Yr* Year.

^a^Gestational weight gain is divided into ‘low’, ‘normal’ and ‘high’ groups according to the guidelines established by the Institute of Medicine (IOM) of the National Research Council in 2009.

Children born to mothers with a BMI of ≥25 kg/m^2^ in early pregnancy had a 1.2 (95% CI: 1.1–1.3) times higher risk of ALL than those born to mothers of normal weight, whereas no statistically significant association was observed between maternal underweight and ALL in the offspring (SIR = 1.1, 95% CI: 0.9–1.4, Table 2). When stratified by sex, no increased risk was observed in sons whose mothers were overweight or obese (SIR = 1.0, 95% CI: 0.9–1.1), whereas daughters born to mothers with a BMI of ≥25 kg/m^2^ had a 40% (SIR = 1.4, 95% CI: 20–60%) higher risk than those born to mothers with normal weight (Table 2). To statistically check the sex difference, we performed the interaction test using Cox proportional hazards model and observed a multiplicative interaction between sex and maternal overweight or obesity (*P* = 0.02 for interaction, Supplementary eTable 2 in Supplement).

**Table 2:** Maternal weight during pregnancy and risk of childhood acute lymphoblastic leukemia by child’s sex.

Exposure	Pregnancies, No.	Total		Girls		Boys	
		ALL, No.	SIR (95% CI)	ALL, No.	SIR (95% CI)	ALL, No.	SIR (95% CI)
Maternal BMI in early pregnancy (kg/m^2^)^a,b^							
<18.5	105,899	60	1.1 (0.9–1.4)	26	1.1 (0.7–1.6)	34	1.1 (0.8–1.6)
18.5–24.9	1,911,793	891	Reference	376	Reference	515	Reference
≥25	943,743	437	**1.2** (1.1–1.3)	214	**1.4** (1.2–1.6)	223	1.0 (0.9–1.1)
25–29.9	662,105	311	**1.1** (1.0–1.3)	151	**1.4** (1.1–1.6)	160	1.0 (0.8–1.1)
≥30	281,638	126	**1.2** (1.0–1.5)	63	**1.5** (1.2–2.0)	63	1.0 (0.8–1.3)
Maternal BMI before delivery (kg/m^2^)^a,c^							
<22.5	58,864	31	1.0 (0.7–1.4)	13	1.0 (0.5–1.7)	18	1.0 (0.6–1.6)
22.5–28.9	818,979	417	Reference	171	Reference	246	Reference
≥29	585,298	270	1.1 (1.0–1.2)	132	**1.4** (1.1–1.6)	138	0.9 (0.8–1.1)
29–33.9	422,271	197	1.1 (0.9–1.2)	98	**1.3** (1.1–1.6)	99	0.9 (0.7–1.1)
≥34	163,027	73	1.2 (1.0–1.5)	34	**1.5** (1.0–2.0)	39	1.1 (0.8–1.5)
Maternal weight in early pregnancy (kg)^a,b,d^							
<50	115,770	47	0.8 (0.6–1.1)	21	0.8 (0.5–1.3)	26	0.8 (0.5–1.2)
50–59	811,933	375	Reference	161	Reference	214	Reference
60–69	1,072,569	512	**1.1** (1.0–1.2)	210	1.1 (1.0–1.3)	302	**1.2** (1.0–1.3)
≥70	961,163	450	**1.2** (1.1–1.4)	221	**1.5** (1.3–1.7)	229	1.1 (0.9–1.2)
70–79	560,086	275	**1.3** (1.1–1.4)	133	**1.5** (1.3–1.8)	142	1.1 (0.9–1.3)
≥80	401,077	175	**1.2** (1.0–1.4)	88	**1.5** (1.2–1.8)	87	1.0 (0.8–1.3)
Maternal weight before delivery (kg)^a,c,d^							
<62	80,552	38	1.0 (0.7–1.3)	16	0.9 (0.5–1.5)	22	1.0 (0.6–1.5)
62–71	359,047	176	Reference	76	Reference	100	Reference
72–81	488,672	244	1.1 (1.0–1.2)	96	1.1 (0.9–1.3)	148	1.1 (0.9–1.3)
≥82	534,870	248	**1.2** (1.1–1.4)	121	**1.5** (1.2–1.7)	127	1.0 (0.9–1.2)
82–91	306,162	153	**1.2** (1.0–1.4)	72	**1.4** (1.1–1.8)	81	1.1 (0.8–1.3)
≥92	228,708	95	1.2 (1.0–1.5)	49	**1.5** (1.1–2.0)	46	1.0 (0.7–1.3)
Gestational weight gain category^a,c,e^							
Low	372,255	175	1.0 (0.8–1.1)	78	1.0 (0.8–1.3)	97	1.0 (0.8–1.2)
Normal	561,952	280	Reference	115	Reference	165	Reference
High	528,934	260	1.0 (0.9–1.1)	120	1.1 (0.9–1.3)	140	1.0 (0.8–1.2)
Gestational weight gain (kg)^a,c,e^							
<10	276,995	119	0.9 (0.7–1.0)	55	0.9 (0.6–1.1)	64	0.9 (0.7–1.1)
10–14.9	584,525	307	Reference	136	Reference	171	Reference
≥15	601,621	290	0.9 (0.8–1.1)	123	1.0 (0.8–1.2)	167	0.9 (0.8–1.1)

Bold SIR indicate statistically significant.

*ALL* Acute lymphoblastic leukemia, *BMI* Body mass index, *CI* Confidence interval, *GWG* Gestational weight gain, *SIR* Standardized incidence ratio.

^a^Adjusted for calendar period, maternal age at delivery, diabetes, smoking during pregnancy, region of residence and socioeconomic status, and child’s sex and family history of ALL.

^b^Additionally adjusted for child’s birth weight.

^c^Additionally adjusted for child’s birth order and gestational week.

^d^Additionally adjusted for maternal height.

^e^Additionally adjusted for maternal BMI in early pregnancy.

Similar results were found when the absolute value of maternal weight in early pregnancy was examined for the association with the risk of ALL. The risk of ALL in girls born to mothers weighing ≥70 kg was 1.5 times (95% CI: 1.3–1.7) that of girls born to mothers weighing 50–59 kg. No increased risk was found in boys born to mothers who weighed ≥70 kg (SIR = 1.1, 95% CI: 0.9–1.2, Table 2).

We found similar sex-specific trends when we examined the association between risk of childhood ALL and maternal BMI or weight before delivery. Girls born to mothers with BMI ≥ 29 kg/m^2^ before delivery had a 1.4 (95% CI: 1.1–1.6) times higher risk of ALL compared to girls born to mothers with BMI 22.5–28 kg/m^2^. No such significant associations were found in boys (SIR = 0.9, 95% CI: 0.8–1.1, Table 2).

Girls born to overweight or obese mothers had 1.5-fold risk of ALL diagnosed at age 6–18 years (95% CI: 1.2–1.9, Table 3) compared to girls born to mothers with a normal BMI, whereas this increased risk was 1.2-fold (95% CI: 1.1–1.5) for ALL diagnosed under age 6 years. Compared to girls born to mothers with a normal BMI, girls born to overweight or obese mothers who did not smoke during pregnancy had a 1.3-fold risk of ALL (95% CI: 1.1–1.6), whereas girls born to overweight or obese mothers who smoked during pregnancy had a 1.7-fold risk of ALL (95% CI: 1.2–2.5). The increased risk in girls was consistent when stratified by child’s birth weight and maternal age at delivery. In male offspring, no significantly increased risk was found between maternal overweight or obesity in early pregnancy and the risk of ALL in all strata (SIR = 0.8–1.1).

**Table 3:** Maternal body mass index in early pregnancy and risk of childhood acute lymphoblastic leukemia by child’s sex, age at diagnosis and birth weight, and maternal smoking and age at delivery.

	Maternal BMI in early pregnancy (kg/m^2^)	Pregnancies, No.	Total		Girls		Boys	
			ALL, No.	SIR (95% CI)	ALL, No.	SIR (95% CI)	ALL, No.	SIR (95% CI)
Child’s age at diagnosis (yr)^a,b,c,d^								
0–5	<18.5	105,899	38	1.2 (0.9–1.7)	14	0.9 (0.5–1.5)	24	1.5 (1.0–2.2)
	18.5–24.9	1,911,792	568	Reference	262	Reference	306	Reference
	≥25	943,743	279	1.0 (0.9–1.2)	146	**1.2** (1.1–1.5)	133	0.9 (0.7–1.0)
6–18	<18.5	88,458	22	1.1 (0.7–1.7)	12	1.8 (0.9–3.1)	10	0.8 (0.4–1.4)
	18.5–24.9	1,531,241	323	Reference	114	Reference	209	Reference
	≥25	685,123	158	**1.2** (1.0–1.4)	68	**1.5** (1.2–1.9)	90	1.1 (0.9–1.3)
Child’s birth weight (g)^a,c,d^								
<3500	<18.5	67,928	39	1.2 (0.9–1.7)	16	1.0 (0.6–1.6)	23	1.5 (1.0–2.3)
	18.5–24.9	906,071	377	Reference	192	Reference	185	Reference
	≥25	361,892	148	1.2 (1.0–1.4)	86	**1.4** (1.1–1.8)	62	0.9 (0.7–1.2)
≥3500	<18.5	37,661	21	1.0 (0.6–1.5)	10	1.3 (0.6–2.4)	11	0.8 (0.4–1.4)
	18.5–24.9	1,000,868	512	Reference	183	Reference	329	Reference
	≥25	579,801	288	**1.1** (1.0–1.3)	127	**1.4** (1.1–1.6)	161	1.0 (0.9–1.2)
Maternal smoking during pregnancy^a,b,c^								
Non-smoker	<18.5	78,815	46	1.2 (0.9–1.6)	17	1.0 (0.6–1.5)	29	1.3 (0.9–1.9)
	18.5–24.9	1,626,078	743	Reference	318	Reference	425	Reference
	≥25	809,610	362	**1.1** (1.0–1.3)	177	**1.3** (1.1–1.6)	185	1.0 (0.9–1.1)
Smoker	<18.5	23,411	14	1.3 (0.7–2.1)	9	1.9 (0.9–3.5)	5	0.8 (0.3–1.8)
	18.5–24.9	244,413	119	Reference	49	Reference	70	Reference
	≥25	120,622	68	**1.3** (1.0–1.7)	32	**1.7** (1.2–2.5)	36	1.1 (0.7–1.5)
Maternal age at delivery (yr)^a,b,d^								
<30	<18.5	67,690	37	1.1 (0.8–1.5)	14	1.0 (0.5–1.6)	23	1.2 (0.8–1.8)
	18.5–24.9	923,411	439	Reference	184	Reference	255	Reference
	≥25	418,222	195	1.2 (1.0–1.3)	93	**1.4** (1.1–1.7)	102	1.0 (0.8–1.2)
≥30	<18.5	38,209	23	1.2 (0.8–1.8)	12	1.4 (0.7–2.4)	11	1.0 (0.5–1.9)
	18.5–24.9	988,382	452	Reference	192	Reference	260	Reference
	≥25	525,521	242	**1.2** (1.0–1.3)	121	**1.4** (1.2–1.7)	121	1.0 (0.8–1.2)

Bold SIR indicate statistically significant.

*ALL* Acute lymphoblastic leukemia, *BMI* Body mass index, *CI* Confidence interval, *SIR* Standardized incidence ratio, *Yr* Year.

^a^Adjusted for calendar period, maternal region of residence, socioeconomic status, and diabetes, and child’s sex and family history of ALL.

^b^Additionally adjusted for child’s birth weight.

^c^Additionally adjusted for maternal age at delivery.

^d^Additionally adjusted for maternal smoking during pregnancy.

### Gestational weight gain

The analysis between the association of GWG and childhood ALL was adjusted for maternal BMI before pregnancy. There was a total of 1,463,141 children included. 25.4% were born to mothers with low GWG according to the IOM classification, 38.4% to mothers with normal GWG and 36.2% to mothers with high GWG.

We observed that children born to mothers with high and low GWG did not have a different risk of ALL than those born to mothers with normal GWG (both SIRs = 1.0, Table 2). There was also no difference in the risk of ALL in children of mothers who gained <10 kg, 10–15 kg, or ≥15 kg during pregnancy (Table 2). No significant difference in the risk of ALL was found between different GWG groups when analyses were stratified according to the child’s sex, child’s age at diagnosis of ALL, maternal BMI in early pregnancy, and maternal smoking during pregnancy (Supplementary eTable 3 in Supplement).

### Sensitivity analysis

After excluding children with a family history of ALL, girls born to overweight or obese mothers still had a higher risk of childhood ALL than girls born to mothers with a normal weight (SIR = 1.4, 95% CI: 1.2–1.6, Supplementary eTable 4 in Supplement), whereas the risk for boys remained unchanged (SIR = 1.0, 95% CI: 0.9–1.1).

## Discussion

We conducted a nationwide cohort study of nearly 3 million children. 1388 children were diagnosed with ALL during follow-up, which to our knowledge makes this study the largest to date examining the association between maternal weight characteristics during pregnancy and the risk of ALL in offspring.

Our study demonstrated an association between maternal overweight and obesity and an increased risk of childhood ALL, which is consistent with the findings from a previous cohort study involving 581 ALL cases in Pennsylvania [14]. In that study, a 45% higher risk of ALL was found in children born to moderately and severely obese mothers (BMI ≥ 35 kg/m^2^) than in those born to mothers with normal weight. In our study, we found a 40% increased risk of ALL even in girls born to overweight mothers (BMI 25–29 kg/m^2^). In addition, our study showed that only daughters, not sons, born to overweight or obese mothers had a higher risk of developing ALL. This is the first study to highlight the sex difference in the association between maternal BMI in early pregnancy and the risk of childhood ALL.

Interestingly, sex differences have been reported in many other aspects of ALL. The incidence of ALL has been reported to be higher in boys than in girls [26]. No significant difference in overall ALL mortality between boys and girls was found, but girls with ALL have a higher risk of death than boys compared to the same-sex population [27]. Girls treated for childhood ALL have been reported to have a higher survival rate than boys [28, 29]. Girls also tend to have more treatment-related and infection-related deaths than boys [30, 31]. There are no established explanations for the sex difference in ALL incidences, mortality, and response to treatment. A sex hormone-related or chromosome-related etiology may play a role in the sex difference observed in our study. Elevated levels of circulating estrogen and free cholesterol in obese women may increase fetal estrogen exposure [32–34]. Studies have shown that estrogen exposure downregulates the expression of tumor suppressor gene RASSF (Ras association domain family) in breast cancer [35, 36], thereby abolishing the inhibition of oncogene KRAS [37, 38]. Given that KRAS is one of the common mutated protooncogenes in childhood ALL [39] and RASSF2 knockout mice exhibit hematopoietic abnormalities [40], RASSF2 silencing induced by maternal obesity may contribute to the increased risk of childhood ALL in offspring. However, male offspring may be less susceptible to this mechanism due to the presence of binding sites to Y chromosome-linked transcription factor SRY on RASSF [41]. This may partially explain the sex difference observed in our study.

Another hypothesis posits that the X chromosome carries more immune-regulatory genes [42, 43], which may protect girls from childhood ALL [44]. However, maternal obesity may disrupt immune homeostasis by altering the expression of immune-related genes on the X chromosome through epigenetic mechanisms such as DNA methylation [45]. Nevertheless, Martin et al. found that maternal pre-pregnancy obesity was associated with a significantly higher number of DNA methylation sites in female offspring than in male offspring [46], suggesting that female fetuses may be more susceptible to genomic programming.

Furthermore, obesity is often inherited, and one study also found that the mother’s BMI was more strongly correlated with the daughter’s BMI than the father’s BMI [47], suggesting that daughters of obese mothers are more likely to have obesity themselves. There is a lack of direct evidence to support a causal association between childhood obesity and childhood leukemia. Indirect evidence from studies on other relevant populations (such as the increased risk of leukemia in obese adults [48], the increased risk of childhood ALL in children born with high birth weight [14], and the worse prognosis of obese pediatric leukemic patients [49]), as well as some biological clues [50, 51], may support a potential association. This heritable susceptibility to obesity and the potential association between childhood obesity and childhood ALL may partially explain the observed sex-specific association. However, current knowledge regarding in-utero estrogen levels, chromosome gene expression, and the heritability of obesity might be insufficient to fully elucidate our findings. Future studies are warranted to delve deeper into the estrogen-related or chromosome-related biological mechanisms. Additionally, longitudinal studies on obesity-related environmental factors and mediation analyses using childhood BMI and in-utero estrogen levels are warranted to address confounding factors and explore potential mediators.

In the analysis for risk of childhood ALL by maternal BMI in early pregnancy stratified by child’s birth weight, we found consistent association across the two birth weight strata. This indicates that this association was unlikely to be mediated by child’s birth weight. When stratified by child’s age at diagnosis, a higher risk of ALL associated with maternal overweight or obesity was observed among children aged 6–18 years compared to those under 6 years of age. However, due to the limited number of ALL cases in each age stratum, the difference did not reach statistical significance, and no further statistical test was performed. Nevertheless, our finding aligns with a growing body of evidence linking maternal obesity to an increased risk of various adult-onset diseases in offspring, including coronary heart disease, stroke, type 2 diabetes, and colorectal cancer [52–54]. This suggests a potential long-term and profound association between maternal obesity and offspring health, including cancer development, which warrants further investigation.

Moreover, the association between maternal overweight/obesity and childhood ALL was stronger in daughters whose mothers smoked during pregnancy compared to those whose mothers did not. Maternal smoking is a known risk factor for adverse pregnancy outcomes [55, 56]. A case-only study in California found that common ALL-associated gene deletions were detectable in children of smoking mothers [57]. These suggest that exposure to maternal smoking may be a modifier in the association of maternal overweight or obesity and ALL in offspring. However, due to the small number of ALL patients in these strata in our study, the confidence intervals overlapped with each other, indicating that further studies are warranted to confirm these observations.

The association between GWG and childhood ALL was examined in our study and no significant difference in ALL risk was found in the different weight gain groups in either male or female offspring. Our findings are consistent with two prior case-control studies and a registry-based cohort study of a limited number of cancer cases [14, 16, 17]. A meta-analysis also found no significant association between GWG and the risk of any childhood cancer although their data for ALL was insufficient for meta-analysis [15]. This may come as a relief to mothers who gain excess weight during pregnancy because, at least so far, excess GWG does not appear to be associated with an increased risk of childhood malignancies. More than half of the children were excluded in studying GWG due to lack of information on maternal weight before delivery. This was because the Medical Birth Register did not systematically collect data on maternal weight before delivery from 1993. The database quality analysis report states that these missing data obviously affect the prevalence estimates, but usually have little effect on risk estimates because the lack of information seem to be random [20, 58, 59]. We compared the included and excluded children and found that they were comparable in terms of basic characteristics (Supplementary eTable 5 in Supplement). This may show that our missing data are unrelated to our outcome of interest, ALL, therefore, a bias due to missing data seems to be less likely.

The strength of our study is the use of the high-quality population-based registers in Sweden, which are among the largest family-cancer and birth datasets in the world. By linking data from several registries, we could obtain detailed and accurate information on individual’s cancer status [60], as well as potential confounding factors, such as maternal disease, area of residence, and socioeconomic status [61], thereby mitigating information bias by adjustment for potential confounders. In addition, we converted the child’s family history of ALL into dynamic variables to resemble a more realistic setting. However, we did not have information on children’s weight and height for months and years after birth, especially before the cancer diagnosis. Therefore, the probability of residual confounding could not be ruled out.

In conclusion, our study found that maternal overweight and obesity in early pregnancy is an important risk factor for childhood ALL only in girls, whereas excess or deficient GWG was not associated with an increased risk of ALL risk. Our findings update the knowledge of risk factors for childhood ALL and assist medical counselors in providing weight management advice to expectant and pregnant mothers. The mechanism behind this mother-daughter association stimulates further research on sex hormone/chromosome-related etiology of ALL.

## Supplementary information

Supplemental material

## Data Availability

The national registry data used in this study cannot be made publicly available by the study authors. However, explanations of the data and the e-mail address of the contact person for accessing the data are available on the following links: https://www.socialstyrelsen.se/en/statistics-and-data/registers/national-medical-birth-register/, https://www.socialstyrelsen.se/en/statistics-and-data/registers/national-cancer-register/, https://www.socialstyrelsen.se/en/statistics-and-data/registers/national-prescribed-drug-register/, https://www.socialstyrelsen.se/en/statistics-and-data/registers/national-patient-register/.
