# Supplementary material for: Maternal weight during pregnancy and risk of childhood acute lymphoblastic leukemia in offspring
Source: Leukemia. 2025 Jan 26;39(3):590–8. doi: 10.1038/s41375-025-02517-6 (PMC11879861; doi:10.1038/s41375-025-02517-6)
Supplement: Supplementary file 1 — Supplemental material [file 41375_2025_2517_MOESM1_ESM.pdf]

Supplemental Data

eMethods Descriptions of Swedish national registers

eReferences

eTable 1. Inclusion codes used in Swedish Cancer Registry and Patient Register

eTable 2. Interaction between maternal body mass index in early pregnancy and child's sex for risk of childhood acute lymphoblastic leukemia

eTable 3. Maternal gestational weight gain and risk of childhood acute lymphoblastic leukemia by child's sex and maternal BMI in early pregnancy and smoking

eTable 4. Sensitivity analysis for maternal body mass index in early pregnancy and risk of childhood acute lymphoblastic leukemia by child's sex

eTable 5. Distribution of child and maternal characteristics in population with and without information on gestational weight gain

eMethods Descriptions of Swedish national registers

Swedish Medical Birth Register

The Medical Birth Register was established in 1973 and is updated annually.[1] It covers all births in Sweden, with over 4 million birth records by 2018.[2] It contains high-quality information on pregnancies and newborns reported by maternity, obstetric and newborn care services.[1] The data includes maternal data (including height, weight, self-reported obstetric history, smoking habits, etc.) and newborn data (including sex, gestational age, birth weight, etc.).

Swedish Family-Cancer Database

This database links individuals using unique personal identification numbers replaced by pseudonymized serial numbers in several national health registries, including the Cancer Registry, the National Patient Register, the National Population Register, the Multi-generation Register, the Cause of Death Register, and national censuses.[3] It is one of the largest family-cancer databases in the world.

National Cancer Register

The National Cancer Register was founded in 1958 and contains information on primary tumors diagnosed by health care providers in Sweden, including date at diagnosis and tumor characteristics (location and morphology).[4] Its completeness is estimated at 96%.[5] Hematological cancers are identified using the International Classification of Diseases, 8th Revision (ICD-8) and Pathological Anatomical Diagnosis (PAD) codes.

National Patient Register

The National Patient Register has collected nationwide data on diagnoses of diseases from all private and public hospitals since 1964 and visits to specialist physicians in Sweden since 2001.[6, 7] The data includes administrative data, diagnoses and procedures. Diseases such as diabetes and birth defects can be extracted using ICD-8 during 1969-1986, ICD-9 during 1987-1996 and ICD-10 since 1997.

Other nationwide registers

Longitudinal demographic information, such as birth dates, death dates, migration, and employment data, can be obtained from the National Population Register, the Cause of Death Register, and national censuses.[8, 9] We retrieved the pedigree of each individual and derived their first-degree relatives (FDRs including parents, siblings, and children) from the Multi-Generation Register.

eReferences

1. Cnattingius S, Kallen K, Sandstrom A, Rydberg H, Mansson H, Stephansson O, et al. The Swedish medical birth register during five decades: documentation of the content and quality of the register. Eur J Epidemiol. 2023;38(1):109-20.
2. Socialstyrelsen. Statistical register's production and quality_National Medical Birth Register. 2021. https://www.socialstyrelsen.se/globalassets/sharepoint-dokument/dokument-webb/ovrigt/production-and-quality-mfr.pdf.
3. Hemminki K, Ji J, Brandt A, Mousavi SM, Sundquist J. The Swedish Family-Cancer Database 2009: prospects for histology-specific and immigrant studies. Int J Cancer. 2010;126(10):2259-67.
4. Socialstyrelsen. Statistical register's production and quality_National Cancer Register. 2023. https://www.socialstyrelsen.se/globalassets/sharepoint-dokument/dokument-webb/ovrigt/production-and-quality-can.pdf.
5. Barlow L, Westergren K, Holmberg L, Talbäck M. The completeness of the Swedish Cancer Register: a sample survey for year 1998. Acta Oncol. 2009;48(1):27-33.
6. Socialstyrelsen. Statistical register's production and quality_National Patient Register. 2023. https://www.socialstyrelsen.se/globalassets/sharepoint-dokument/dokument-webb/statistik/production-and-quality-of-the-patient-register.pdf.
7. Ludvigsson JF, Andersson E, Ekbom A, Feychting M, Kim JL, Reuterwall C, et al. External review and validation of the Swedish national inpatient register. BMC Public Health. 2011;11:450.
8. Ludvigsson JF, Almqvist C, Bonamy AK, Ljung R, Michaelsson K, Neovius M, et al. Registers of the Swedish total population and their use in medical research. Eur J Epidemiol. 2016;31(2):125-36.
9. Brooke HL, Talback M, Hornblad J, Johansson LA, Ludvigsson JF, Druid H, et al. The Swedish cause of death register. Eur J Epidemiol. 2017;32(9):765-73.

eTable 1. Inclusion codes used in Swedish Cancer Registry and Patient Register

Swedish Registers	Diseases	Codes[a]	
**Cancer Registry**	**ALL**	ICD-8	204.0
		ICD-O	C910
		PAD	206, 296, 996
		ICD-O	9821.3, 9820.3, 8000.3
**Patient Register**	**Diabetes**	ICD-8	250
		ICD-9	250, 648A, 648W
		ICD-10	E10, E11, O240, O241, O243, O244, O249
	**Birth defects**	ICD-8	740-759
		ICD-9	740-759
		ICD-10	Q00-Q99

ALL=Acute lymphoblastic leukemia; ICD=International Classification of Diseases; ICD-O=International Classification of Diseases for Oncology, revision 2; PAD=Pathological Anatomical Diagnosis

[a] All codes are based on Swedish versions.

eTable 2. Interaction between maternal body mass index in early pregnancy and child's sex for risk of childhood acute lymphoblastic leukemia

Maternal BMI in early pregnancy (kg/m^2)	Pregnancies, No.	Girls		Boys		P for interaction
		ALL, No.	HR[a] (95%CI)	ALL, No.	HR[a] (95%CI)	
<18.5	105 899	26	1.2 (0.8-1.8)	34	1.2 (0.8-1.7)	0.92
18.5-24.9	1 911 793	376	Reference	515	Reference	
≥25	943 743	214	**1.3** (1.1-1.5)	223	1.0 (0.8-1.1)	**0.02**

ALL=Acute lymphoblastic leukemia; BMI=Body mass index; CI=Confidence interval; HR=Hazard ratio.
[a] Adjusted for calendar period, maternal region of residence, socioeconomic status, age at delivery, diabetes, and smoking during pregnancy, and child's birth weight and family history of ALL.

eTable 3. Maternal gestational weight gain and risk of childhood acute lymphoblastic leukemia by child's sex and maternal BMI in early pregnancy and smoking

	GWG	Pregnancies, No.	Total		Girls		Boys	
			ALL, No.	SIR (95%CI)	ALL, No.	SIR (95%CI)	ALL, No.	SIR (95%CI)
**Child's age at diagnosis (yr)[a,b,c]**								
**0-5**	Low	372 254	113	1.1 (0.9-1.3)	54	1.2 (0.9-1.5)	59	0.9 (0.7-1.2)
	Normal	561 952	169	Reference	72	Reference	97	Reference
	High	528 934	167	1.1 (0.9-1.3)	82	1.2 (1.0-1.6)	85	1.0 (0.8-1.2)
**6-18**	Low	305 846	62	0.9 (0.7-1.1)	24	0.8 (0.5-1.2)	38	0.9 (0.6-1.3)
	Normal	469 964	111	Reference	43	Reference	68	Reference
	High	419 506	93	0.9 (0.7-1.1)	38	0.8 (0.6-1.1)	55	1.0 (0.8-1.3)
**Maternal BMI in early pregnancy (kg/m^2)[a,b]**								
**<25**	Low	320 072	152	1.0 (0.8-1.1)	70	1.1 (0.8-1.3)	82	0.9 (0.7-1.1)
	Normal	448 688	230	Reference	91	Reference	139	Reference
	High	272 480	137	1.0 (0.8-1.2)	58	1.1 (0.9-1.5)	79	0.9 (0.7-1.2)
**≥25**	Low	52 183	24	1.3 (0.8-2.0)	9	0.9 (0.4-1.8)	15	1.7 (1.0-2.9)
	Normal	113 264	50	Reference	24	Reference	26	Reference
	High	256 454	124	1.0 (0.9-1.2)	63	1.0 (0.8-1.3)	61	1.1 (0.8-1.4)
**Maternal smoking during pregnancy[a,c]**								
**No**	Low	292 461	137	1.0 (0.8-1.2)	65	1.2 (0.9-1.5)	72	0.9 (0.7-1.1)
	Normal	454 484	222	Reference	87	Reference	135	Reference
	High	430 348	201	1.0 (0.8-1.1)	93	1.1 (0.9-1.3)	108	0.9 (0.7-1.1)
**Yes**	Low	68 604	26	0.7 (0.5-1.1)	10	0.5 (0.3-1.0)	16	1.0 (0.6-1.6)
	Normal	90 344	47	Reference	25	Reference	22	Reference
	High	83 862	51	1.2 (0.9-1.6)	21	0.9 (0.6-1.4)	30	**1.6** (1.1-2.2)

ALL=Acute lymphoblastic leukemia; BMI=Body mass index; CI=Confidence interval; GWG=Gestational weight gain; SIR=Standardized incidence ratio.

[a] Adjusted for calendar period, maternal region of residence, socioeconomic status, age at delivery and diabetes, child's sex, birth order, gestational week at delivery, and family history of ALL.

[b] Additionally adjusted for maternal smoking during pregnancy.

[c] Additionally adjusted for maternal BMI in early pregnancy.

eTable 4. Sensitivity analysis for maternal body mass index in early pregnancy and risk of childhood acute lymphoblastic leukemia by child's sex

Maternal BMI in early pregnancy (kg/m^2)	Pregnancies, No.	Total		Girls		Boys	
		ALL, No.	SIR (95%CI)	ALL, No.	SIR (95%CI)	ALL, No.	SIR (95%CI)
**Excluding children with family history of ALL[a]**							
<18.5	105 851	60	1.1 (0.9-1.4)	26	1.1 (0.7-1.6)	34	1.1 (0.8-1.6)
18.5-24.9	1 910 717	890	Reference	376	Reference	514	Reference
≥25	943 034	435	**1.2** (1.0-1.3)	212	**1.4** (1.2-1.6)	223	1.0 (0.9-1.1)

ALL=Acute lymphoblastic leukemia; BMI=Body mass index; CI=Confidence interval; SIR=Standardized incidence ratio. Bold SIR indicates statistically significant.

[a] Adjusted for calendar period, maternal region of residence, socioeconomic status, age at delivery, diabetes, and smoking during pregnancy, and child's sex and birth weight.

eTable 5. Distribution of child and maternal characteristics in population with and without information on gestational weight gain

	Information on gestational weight gain	
	Available	Not available
Characteristics	No. (%)	No. (%)
**Total**	1 463 141 (49.41)	1 498 294 (50.59)
**Children**		
**Sex**		
Male	752 840 (51.45)	769 499 (51.36)
Female	710 301 (48.55)	728 795 (48.64)
**Birth year**		
1983-1989	521 117 (35.62)	1 (0.00)
1990-1999	285 915 (19.54)	369 914 (24.69)
2000-2009	292 188 (19.97)	562 827 (37.56)
2010-2018	363 921 (24.87)	565 552 (37.75)
**Birth weight (g)**		
<2500	37 096 (2.54)	49 267 (3.29)
2500-3499	619 826 (42.36)	629 702 (42.03)
3500-4499	748 859 (51.18)	760 255 (50.74)
≥4500	53 644 (3.67)	55 572 (3.71)
Missing	3 716 (0.25)	3 498 (0.23)
**Gestational week**		
<37	59 209 (4.05)	75 310 (5.03)
37-41	1 297 525 (88.68)	1 317 647 (87.94)
≥42	105 664 (7.22)	105 097 (7.01)
Missing	743 (0.05)	240 (0.02)
**Birth order**		
1	587 243 (40.14)	557 494 (37.20)
2	478 168 (32.68)	485 416 (32.40)
≥3	278 389 (19.02)	263 668 (17.60)
Missing	119 341 (8.16)	191 716 (12.80)
**First-degree relatives with ALL**		
No	1 461 397 (99.88)	1 496 507 (99.88)
Yes	1 644 (0.11)	1 697 (0.11)
Missing	100 (0.01)	90 (0.01)
**Mothers**		
**Maternal BMI in early pregnancy (kg/m^2)**		
<18.5	66 162 (4.52)	39 737 (2.65)
18.5-24.9	975 078 (66.64)	936 715 (62.52)
25-29.9	298 328 (20.39)	363 777 (24.28)
≥30	123 573 (8.45)	158 065 (10.55)
**Delivery age**		
<25	265 488 (18.15)	191 564 (12.79)
25-34	953 299 (65.15)	969 259 (64.69)
≥35	244 354 (16.70)	337 471 (22.52)
**Smoking during pregnancy**		
No	1 177 293 (80.46)	1 337 210 (89.25)
Yes	242 810 (16.60)	145 636 (9.72)
Missing	43 038 (2.94)	15 448 (1.03)
**Maternal diabetes**		
No	1 439 064 (98.35)	1 469 882 (98.10)
Yes	24 077 (1.65)	28 412 (1.90)

ALL=Acute lymphoblastic leukemia; BMI=Body mass index.
